# Cost-Effectiveness Analysis of a Procalcitonin-Guided Decision Algorithm for Antibiotic Stewardship Using Real-World U.S. Hospital Data

**DOI:** 10.1089/omi.2019.0113

**Published:** 2019-10-04

**Authors:** Anne M. Voermans, Janne C. Mewes, Michael R. Broyles, Lotte M. G. Steuten

**Affiliations:** ^1^ Panaxea B.V., Amsterdam, the Netherlands.; ^2^ Department of Clinical Pharmacy and Laboratory Services, Pocahontas, Five Rivers Medical Center, Arkansas.; ^3^ Office of Health Economics, London, United Kingdom.

**Keywords:** algorithms, procalcitonin, cost-effectiveness, sepsis, antibiotic stewardship, biomarkers, health economics

## Abstract

Medical decision-making is revolutionizing with the introduction of artificial intelligence and machine learning. Yet, traditional algorithms using biomarkers to optimize drug treatment continue to be important and necessary. In this context, early diagnosis and rational antimicrobial therapy of sepsis and lower respiratory tract infections (LRTI) are vital to prevent morbidity and mortality. In this study we report an original cost-effectiveness analysis (CEA) of using a procalcitonin (PCT)-based decision algorithm to guide antibiotic prescription for hospitalized sepsis and LRTI patients versus standard care. We conducted a CEA using a decision-tree model before and after the implementation of PCT-guided antibiotic stewardship (ABS) using real-world U.S. hospital-specific data. The CEA included societal and hospital perspectives with the time horizon covering the length of hospital stay. The main outcomes were average total costs per patient, and numbers of patients with *Clostridium difficile* and antibiotic resistance (ABR) infections. We found that health care with the PCT decision algorithm for hospitalized sepsis and LRTI patients resulted in shorter length of stay, reduced antibiotic use, fewer mechanical ventilation days, and lower numbers of patients with *C. difficile* and ABR infections. The PCT-guided health care resulted in cost savings of $25,611 (49% reduction from standard care) for sepsis and $3630 (23% reduction) for LRTI, on average per patient. In conclusion, the PCT decision algorithm for ABS in sepsis and LRTI might offer cost savings in comparison with standard care in a U.S. hospital context. To the best of our knowledge, this is the first health economic analysis on PCT implementation using U.S. real-world data. We suggest that future CEA studies in other U.S. and worldwide settings are warranted in the current age when PCT and other decision algorithms are increasingly deployed in precision therapeutics and evidence-based medicine.

## Introduction

Sepsis and lower respiratory tract infections (LRTI) cause morbidity and mortality among hospitalized patients (Dellinger et al., 2013). Early diagnosis and appropriate antimicrobial therapy are vital in treatment of these patients (Carlet, 1999). However, overprescribing antibiotics can contribute to antibiotic resistance (ABR) and *Clostridium difficile* infections (CDI) (Schuetz et al., 2011; Wenzel and Edmond, 2000).

Guidance on when to initiate or terminate antibiotic therapy could aid reducing the overuse of antibiotics, and thereby reduce ABR and the number of CDI patients. Procalcitonin (PCT) is a biomarker that is able to provide guidance in clinical decision-making on antibiotic usage (Schuetz et al., 2015). PCT can distinguish bacterial from nonbacterial infections even in early stages of inflammation with good specificity (Póvoa and Salluh, 2012). Typically, within 3–4 h after onset of an inflammatory response PCT is elevated, after which it peaks at 14–25 h. With a half-life of ∼24 h, PCT decreases rapidly when the inflammatory response begins to resolve (Linscheid et al., 2003; Müller et al., 2001). PCT values can thus support clinical decision-making on antibiotic initiation and discontinuation.

PCT-guided antibiotic stewardship (ABS) was found to be safe and contribute to reducing the use of antibiotics (Albrich et al., 2012). However, PCT implementation comes at additional costs for the additional blood tests. Cost-effectiveness analyses (CEAs) for hospitalized sepsis and LRTI patients have shown that net savings in downstream costs offset the increased PCT testing costs (Harrison and Collins, 2015; Heyland et al., 2011; Kip et al., 2015; Mewes et al., 2019), while decreasing antibiotic resource utilization. The earlier CEAs were performed based on both European and U.S. data (Kip et al., 2015; Mewes et al., 2019).

To further support the adoption and uptake of PCT testing in the United States, there is a need to quantify the added value of PCT testing in a U.S. hospital setting in terms of cost-effectiveness. Therefore, this study reports on real-world data of a U.S. hospital to populate a previously published decision-tree model (Mewes et al., 2019) and performs a model-based analysis of the cost-effectiveness of a PCT algorithm versus standard care to guide antibiotic prescription for hospitalized sepsis and LRTI patients in a U.S. hospital setting.

## Materials and Methods

**FIG. 1.**
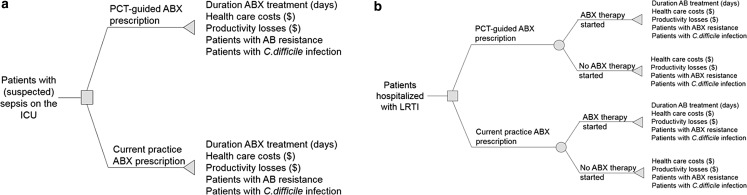
Decision tree for sepsis **(a)** and LRTI **(b)** patients (Mewes et al., 2019). ABX, antibiotics; ICU, intensive care unit; LRTI, lower respiratory tract infections; PCT, procalcitonin.

A previously published decision-tree model (Kip et al., 2015; Mewes et al., 2019) (Fig. 1) was populated with real-world U.S. hospital data. PCT-guided antibiotic use was compared with standard care for sepsis and LRTI patients. In PCT-guided care, an algorithm was used to guide the decision on initiation (LRTI) and discontinuation of (both sepsis and LRTI) antibiotic therapy. Standard care included all usual care, except for the PCT algorithm on antibiotic initiation or discontinuation.

The analyses were conducted from the hospital and societal perspective. The time horizon covered the length of hospital stay. Model analyses for sepsis or LRTI patients were performed separately. For LRTI, hospitalized patients with respiratory infections, chronic obstructive pulmonary disease (COPD), or pneumonia were considered.

### Data collection

Patient data collection was performed in the Five Rivers Medical Center (FRMC), Pocahontas, Arkansas. Research ethics approval was granted by FRMC Medical Executive Committee, Pharmacy & Therapeutics, and Governing Board. Data were collected in two 4-year time periods: 2006–2009 (no PCT testing) and after implementation of PCT testing, 2010–2014. After PCT implementation, PCT testing was a prechecked field on the admission order set for suspected infection.

For each individual patient the diagnosis-related group (DRG) (septicemia or severe sepsis/respiratory infections and inflammations/COPD/pneumonia and pleurisy), age, sex, CDI (Y/N), mortality (Y/N), general ward length of stay (LOS), and antibiotic days of therapy (DOT) were reported. DOTs classify antibiotic days based on patient-level exposure and were defined as the number of days a patient was on an antibiotic therapy, assuming appropriate dosing. Multiple antibiotics were counted as multiple DOTs. For sepsis patients the intensive care unit (ICU) LOS was recorded as well. For each year of data collection, the charge per DOT in that year was noted. For both time periods, all laboratory tests and associated costs were recorded on the FRMC population level.

### Model inputs

Where available, data from the FRMC were used. When unavailable, values were taken from the previous study (Mewes et al., 2019), in which a systematic literature review was conducted. Literature estimates from U.S. studies were prioritized for inclusion.

CDI prevalence was determined by calculating the average annual CDI rates in the FRMC. The number of reported CDI patients was divided over the total number of patients in the designated group.

Initial prevalence of ABR infections was determined using resistance data of the U.S. population. Prevalence of ABR infections was 21.7% in sepsis and 22.2% in LRTI patients (The Center for Disease Dynamics, Economics and Policy, 2017). The reduction of ABR infections in the PCT testing period for each group was estimated based on the incremental reduction in antibiotic use. As the percentage of reduction in antibiotic days was correlated with a reduction of 3.2% of the ABR rate (Chastre et al., 2003; van der Maas et al., 2015; Singh et al., 2000), this rate was multiplied with the reduction in antibiotic days found from the FRMC database.

Resource use consisted of the hospital stay (in the general ward and ICU), treatment (mechanical ventilation [MV] and antibiotics), laboratory analyses (blood cultures, PCT tests, and additional tests), and additional resources for CDI and ABR. The latter consisted of isolation, additional blood tests, and extended LOS. Extended LOS for sepsis and LRTI, respectively, were 2.8 and 2.8 days for CDI and 4.6 and 8.1 days for ABR. The LOS equals the time the patient cannot work and thus incurs productivity losses.

For the number of days on MV (applicable to sepsis and LRTI patients on the ICU) and ICU LOS (applicable to LRTI) model inputs were calculated based on ratios from literature (Bishop et al., 2014) combined with the absolute LOS from the FRMC database. MV days were calculated as follows:

\begin{align*}
{ \rm { M } } { { \rm { V } } _ { { \rm { days , \;FRMC } } } } { \rm { = } } { \frac { { \rm { M } } { { \rm { V } } _ { { \rm { days , lit \; } } } } }  { { \rm { LO } } { { \rm { S } } _ { { \rm { total , \;lit } } } } } } { \rm { LO } } { { \rm { S } } _ { { \rm { total , \;FRMC } } } } ,
\end{align*}

where MV_days,lit_ is the number of days on MV reported in literature (Bishop et al., 2014); LOS_total,lit_, the total LOS reported in literature (Bishop et al., 2014), and LOS_total,FRMC_, the total LOS from the FRMC database.

ICU LOS was calculated as follows:

\begin{align*}
{ \rm { LO } } { { \rm { S } } _ { { \rm { ICU , \;FRMC } } } } { \rm { = } } { \frac { { \rm { LO } } { { \rm { S } } _ { { \rm { ICU , lit } } } } }  { { \rm { LO } } { { \rm { S } } _ { { \rm { gw , \;lit } } } } } } { \rm { LO } } { { \rm { S } } _ { { \rm { gw , FRMC } } } } ,
\end{align*}

where LOS_ICU,lit_ is the ICU LOS reported in literature (Bishop et al., 2014); LOS_gw,lit_, the general ward LOS reported in literature (Bishop et al., 2014); and LOS_gw,FRMC_, the general ward LOS from the FRMC database. Table 1 lists all resource use.

**Table 1.** Resource Use in the Five Rivers Medical Center in 2006–2009 (No Procalcitonin) and 2010–2014 (Procalcitonin)

	*Sepsis*		*LRTI*		
	*No PCT*	*PCT*	*No PCT*	*PCT*	*Source*
Hospitalization					
Patients requiring ICU admission (%)	100	100	10.5^a^	10.5^a^	FRMC; Albrich et al. (2012)
LOS general ward	2.9	5.3	3.8	3.3	FRMC
LOS ICU	15.0	4.5	7.9^b^	5.4^b^	FRMC; Bishop et al. (2014)
Total LOS	17.9	9.8	11.7	8.7	FRMC
Treatment					
Patient requiring MV (%)	100	100	10.5	10.5	Bishop et al. (2014)
Days on MV	5.4^b^	2.5^b^	1.17^b^	0.85^b^	FRMC; Bishop et al. (2014)
Patients prescribed antibiotics (%)	100	100	87.7^a^	75.4^a^	FRMC; Schuetz et al. (2009)
Antibiotic DOT	22.8	10.3	15.2	9.4	FRMC
Laboratory analyses					
Patients in whom blood culture was taken (%)	97.5	61.4	97.5	61.4	Müller et al. (2010)
Number of blood cultures taken	1.84	1.16	1.84	1.16	FRMC
Patients with blood culture taken diagnosed as having sepsis (%)	8.18	8.18	N/A	N/A	Shapiro et al. (2008)
Number of laboratory tests	40	28	40	28	FRMC
Number of PCT tests	0	3.1	0	3.1	FRMC
CDI					
Additional LOS general ward owing to CDI	2.8	2.8	2.8	2.8	FRMC
Number of CDI tests per day	1.2	1.2	1.2	1.2	FRMC
ABR					
Additional LOS general ward owing to ABR	4.6	4.6	8.1	8.1	FRMC

^a^ Based on literature.

^b^ Based on literature ratio with FRMC data, see explanation in the Materials and Methods section.

ABR, antibiotic resistance; CDI, *Clostridium difficile* infections; DOT, days of therapy; FRMC, Five Rivers Medical Center; ICU, intensive care unit; LOS, length of stay; LRTI, lower respiratory tract infections; MV, mechanical ventilation; PCT, procalcitonin.

Costs categories included hospital costs, treatment costs, laboratory analyses and productivity losses. All costs were inflated to 2019 U.S. dollars. All costs obtained from the FRMC database were expressed in patient charges. Discounting was not applicable as the time horizon of the model was shorter than 1 year. Table 2 lists all cost inputs.

**Table 2.** Unit Costs (Identical for Sepsis and Lower Respiratory Tract Infections)

	*Unit costs*	*Source*
Hospitalization		
General ward per day	$1304.75	Balk et al. (2017)
ICU per day	$1944.06	The Henry J. Kaiser Family Foundation (2017)
Isolation per day	$51.34	FRMC
Treatment		
MV per day	$1078.24	Centres for Medicare and Medicaid Services (2017)
Antibiotics per DOT	$174.02	FRMC
Laboratory analyses		
Blood culture	$55.41	FRMC
Other laboratory tests without PCT implementation	$78.98	FRMC
Other laboratory tests with PCT implementation	$79.21	FRMC
PCT test	$95.81	FRMC
CDI test	$93.50	FRMC
Productivity losses		
Working hours per day	8	Neumann et al. (2016)
Productivity losses per hour	$21.77	Neumann et al. (2016)

### Analysis

The models for sepsis and LRTI presumed two treatment pathways to which costs were assigned: standard care and PCT-guided ABS. Costs were calculated by multiplying volumes with unit costs. Population-level costs were assessed by multiplication of the annual average number of hospitalized sepsis or LRTI patients with the expected average total costs per strategy. Incremental costs were determined by subtracting costs for standard care from the costs for the PCT-guided care strategy.

The incremental cost-effectiveness ratio (ICER) for costs per DOT avoided was calculated by dividing the incremental costs by the incremental DOTs. The ICER of costs per ABR patient avoided and per CDI patient avoided was analyzed by dividing the incremental costs by the incremental number of patients with ABR and CDI, respectively.

### Sensitivity analysis

A one-way sensitivity analysis was performed to assess the robustness of the model results and to identify the key cost drivers. Each individual parameter was varied by ±25%, whereas other parameters remained at their base case value.

## Results

Patient characteristics during the period before and after implementation of PCT-guided antibiotic therapy are given in Table 3. The average number of annual hospitalizations owing to sepsis and LRTI were 13 and 202, respectively.

**Table 3.** Characteristics of Patients Hospitalized for Either Sepsis or Lower Respiratory Tract Infections in the Five Rivers Medical Center in 2006–2009 (No Procalcitonin) and 2010–2014 (Procalcitonin)

	*Sepsis*		*LRTI*	
	*No PCT*	*PCT*	*No PCT*	*PCT*
Patients (*n*)	13	90	755	860
Age (years) (mean ± SD)	73.4 ± 10.8	71.6 ± 17.0	71.5 ± 16.0	71.9 ± 16.3
Male (%)	61.5	52.2	45.2	44.2

### Sepsis

In the period after PCT implementation, a reduction of 12.5 DOTs, 10.5 ICU days, and 12 laboratory tests, and an increase of 2.4 general ward days per patient were found. Only considering health care costs, total average incremental costs after PCT implementation were −$24,187 per sepsis patient and −$311,404 for the whole sepsis population in the FRMC. Including productivity losses, total incremental costs after PCT implementation were −$25,611 per sepsis patient and $329,747 for the whole sepsis patient population in the FRMC, indicating cost savings. Total costs were reduced by 49.2% compared with standard care.

On the patient population level, it was estimated that the number of ABR patients was reduced by 8.0% after PCT implementation. The ICER was −$2049 and −$1464 per DOT and per ABR patient avoided, respectively, in comparison with standard care. As no CDI patients were reported for sepsis in the FRMC during the study period, no results were available on that subject (Table 4).

**Table 4.** Effectiveness and Cost Outcomes for Patients Hospitalized for Either Sepsis or Lower Respiratory Tract Infections in the Five Rivers Medical Center in 2006–2009 (No Procalcitonin) and 2010–2014 (Procalcitonin)

	*Outcome*	*No PCT*	*PCT*	*Difference*
Sepsis	Effectiveness measures			
	Antibiotic DOT	22.8	10.3	−12.5
	ABR patients	2.80	2.57	−0.23
	CDI patients	0	0	0
	Costs^a^			
	Hospitalization	$32,944.68	$15,663.45	−$17,281.23
	Antibiotics	$3967.66	$1792.41	−$2172.25
	MV	$5809.45	$2736.79	−$3072.66
	Laboratory analyses^b^	$4374.43	$2997.35	−$1377.08
	Additional costs ABR infection			
	Per ABR patient	$7674.38	$6942.26	−$732.12
	Per sepsis patient	$1667.55	$1387.06	−$280.49
	Additional costs CDI			
	Per CDI patient	$0.00	$0.00	$0.00
	Per sepsis patient	$0.00	$0.00	$0.00
	Productivity losses	$3291.54	$1.866.83	−$1424.71
	Average total costs			
	Per sepsis patient	$52,055.30	$26,433.88	−$25,611.42
	Per sepsis patient population	$670,211.97	$340,464.96	−$329,747.01
LRTI	Effectiveness measures			
	Antibiotic DOT	15.2	9.4	−5.8
	ABR patients	39.30	32.52	−6.78
	CDI patients	4.69	1.23	−3.46
	Costs^a^			
	Hospitalization	$6562.93	$5417.14	−$1145.79
	Antibiotics	$2319.76	$1233.38	−$1086.37
	MV	$132.94	$96.77	−$36.17
	Laboratory analyses^b^	$3258.61	$2554.36	−$704.25
	Additional costs ABR infection			
	Per ABR patient	$11,723.47	$11,665.40	−$58.08
	Per LRTI patient	$2282.22	$1879.21	−$403.01
	Additional costs CDI			
	Per CDI patient	$3909.25	$3909.25	$0.00
	Per LRTI patient	$90.85	$23.88	−$66.98
	Productivity losses	$1.091.54	$904.53	−$187.01
	Average total costs			
	Per LRTI patient	$15,738.54	$12.109.26	−$3629.58
	Per LRTI patient population	$3,177,279.09	$2,444,557.75	−$732,721.34

^a^ Average per patient, unless indicated otherwise.

^b^ Including PCT tests in the PCT group.

### Lower respiratory tract infections

In the period after PCT implementation, a reduction of 5.8 DOTs, a reduction of 0.5 days on the regular ward and 2.4 days at the ICU, and 12 fewer laboratory tests taken per patient were found. Only considering health care costs, total average incremental costs after PCT implementation were −$3423 per LRTI patient and −$694,969 for the whole LRTI patient population in the FRMC. Including productivity losses, total incremental costs after PCT implementation were −$3630 per LRTI patient and −$732,721 for the whole LRTI patient population in the FRMC, indicating cost savings. Total costs were reduced by 23.0% compared with standard care.

On the patient population level, it was estimated that the numbers of CDI and ABR patients were reduced by 73.7% and 17.2%, respectively, after implementing PCT-guided antibiotic therapy. The ICER was −$626, −$108,092, and −$211,846 per DOT avoided, per ABR patient avoided, and per CDI patient avoided, respectively (Table 4).

### Sensitivity analysis

**FIG. 2.**
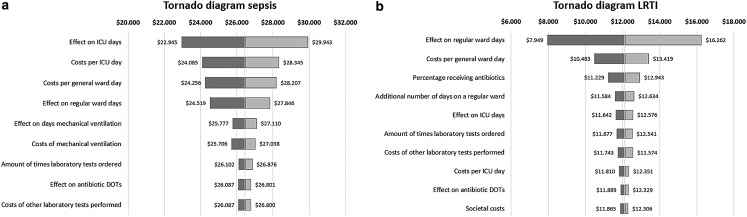
Tornado diagram showing the results of the one-way sensitivity analyses on the total average costs per sepsis **(a)** and LRTI **(b)** patient in comparison with the base case values of the main analysis. DOT, days of therapy.

The one-way sensitivity analysis showed the sepsis population results were most sensitive to (1) the effect on ICU days, (2) the costs per ICU day, and (3) the costs per general ward day (Fig. 2a). For LRTI these were (1) the effect on general ward days, (2) the cost per general ward day, and (3) the percentage of patients receiving antibiotics (Fig. 2b).

## Discussion

Medical decision-making is revolutionizing with the introduction of artificial intelligence and machine learning. Yet, traditional algorithms using biomarkers to optimize drug treatment continue to be important and necessary (Grapov et al., 2018). The objective of this study was to perform model-based analyses of the cost-effectiveness of a PCT algorithm versus standard care to guide ABS for sepsis and LRTI patients in a U.S. hospital setting. To the best of our knowledge, this is the first health economic analysis on PCT implementation using U.S. real-world data.

The total incremental cost per patient was −$25,611 and −$3630 for sepsis and LRTI, respectively. PCT implementation was therefore cost saving. The cost savings were mainly driven by the reduction in LOS for both groups. General ward LOS for sepsis increased with PCT implementation, whereas total LOS was reduced, as the ICU LOS was decreased substantially. Furthermore, the PCT implementation resulted in a shorter duration of antibiotic therapy.

Estimated reductions in numbers of ABR patients were −8.0% and −17.2% for sepsis and LRTI, respectively. The estimated reduction in number of CDI patients was −73.7% for LRTI. As no CDI patients were reported for sepsis in the FRMC during the study period, no results were available on that subject.

Mewes et al. (2019) reported total costs per patient for standard care of $43,430 and $16,218 and for PCT-guided ABS of $32,120 and $13,351 for sepsis and LRTI, respectively. They reported cost savings of $11,311 and $2867 per sepsis and LRTI patient, respectively. The results found in this study were comparable with these. Cost savings after PCT implementation in the FRMC found here were higher.

When input parameters for the model were not available from the real-world hospital data of the FRMC, literature values from the previously published U.S.-specific model were used (Mewes et al., 2019). However, the sensitivity analyses showed the parameter most influencing the total costs was the effect on hospitalization days, which is a parameter for which data were available from the FRMC.

The costs that were used in the model were the patient charges of the FRMC. These do not reflect reimbursement fees and might therefore not be directly comparable with those reported in other publications.

A limitation of this study was the low number of patients included, especially in the usual care period for sepsis. Because of the relatively low number of patients, the uncertainty on the statistical significance should be considered. Still, our results were in line with previously published studies on larger populations. The relatively low number of sepsis patients in the no-PCT period was likely because of the U.S. DRG system. In the no-PCT period, clinicians were more reserved on sepsis coding because of more conservative coding criteria, definitions, and reimbursements and were more likely to only code septic shock as sepsis (Gohil et al., 2016).

In before and after analyses, possible uncertainty on what has caused the effects shown in the results should be noted. The possibility exists that medical staff were more alert to antibiotic-related events after PCT implementation. Considering the FRMC sepsis and LRTI treatment policy after PCT implementation, cost savings were achieved in a setting with strong PCT algorithm enforcement.

In our analysis mortality was not included, as the number of patients in this study was deemed too low to extract reliable data on mortality for these populations. Significant effects of PCT use on mortality have not been reported in the literature (Annane et al., 2013; Deliberato et al., 2013). However, it is expected that mortality could potentially be reduced by PCT-guided antibiotic care in the hospital setting (Broyles, 2017).

Moreover, our analysis did not include quality-adjusted life-years. For future research it is recommended to take into account long-term impact of PCT-guided ABS including the patients' health-related quality of life.

As decision algorithms are increasingly used in guiding decision-making, future research should be conducted on their cost-effectiveness in other real-world settings in the United States and outside.

In conclusion, our model-based analyses showed PCT-guided ABS to result in decreased average costs per patient for sepsis and LRTI in a U.S. hospital setting using real-world data. Treatment costs and productivity losses were reduced. In addition, PCT-guided ABS led to a shorter LOS and lower numbers of patients with ABR and CDI.

## Abbreviations Used

ABR: antibiotic resistance
ABS: antibiotic stewardship
ABX: antibiotics
CDI: *Clostridium difficile* infections
CEA: cost-effectiveness analysis
COPD: chronic obstructive pulmonary disease
DOT: days of therapy
DRG: diagnosis-related group
FRMC: Five Rivers Medical Center
ICER: incremental cost-effectiveness ratio
ICU: intensive care unit
LOS: length of stay
LRTI: lower respiratory tract infections
MV: mechanical ventilation
PCT: procalcitonin

## References

[B1] Albrich W, Dusemund F, Bucher B, et al. (2012). Effectiveness and safety of procalcitonin-guided antibiotic therapy in lower respiratory tract infections in real life: An international, multicenter poststudy survey (ProREAL). Arch Intern Med 172, 715–722 22782201 10.1001/archinternmed.2012.770

[B2] Annane D, Maxime V, Faller J, et al. (2013). Procalcitonin levels to guide antibiotic therapy in adults with non-microbiologically proven apparent severe sepsis: A randomised controlled trial. BMJ Open 3, e002186 10.1136/bmjopen-2012-002186 PMC3586059 23418298

[B3] Balk R, Kadri S, Cao Z, Robinson S, Lipkin C, and Bozette S (2017). Effect of procalcitonin testing on health-care utilization and costs in critically ill patients in the United States. Chest 151, 23–33 27568580 10.1016/j.chest.2016.06.046 PMC6026224

[B4] Bishop B, Bon J, Trienski T, Pasquale T, Martin B, and File TJ (2014). Effect of introducing procalcitonin on antimicrobial therapy duration in patients with sepsis and/or pneumonia in the intensive care unit. Ann Pharmacother 48, 577–583 24519479 10.1177/1060028014520957

[B5] Broyles M (2017). Impact of procalcitonin-guided antibiotic management on antibiotic exposure and outcomes: Real-world evidence. Open Forum Infect Dis 4, ofx213 29164170 10.1093/ofid/ofx213 PMC5695623

[B6] Carlet J (1999). Rapid diagnostic methods in the detection of sepsis. Infect Dis Clin North Am 13, 483–494 10340179 10.1016/s0891-5520(05)70087-8

[B7] Centres for Medicare and Medicaid Services. (2017). DMEPOS fee schedule Baltimore: CMS. https://www.cms.gov/Medicare/Medicare-Fee-for-Service-Payment/DMEPOS FeeSched/DMEPOS-Fee-Schedule.html Accessed 63, 2019

[B8] Chastre J, Wolff M, Fagon J, et al. (2003). Comparison of 8 vs 15 days of antibiotic therapy for ventilator-associated pneumonia in adults: A randomized trial. JAMA 29, 2588–2598 10.1001/jama.290.19.2588 14625336

[B9] Deliberato R, Marra A, Sanches P, et al. (2013). Clinical and economic impact of procalcitonin to shorten antimicrobial therapy in septic patients with proven bacterial infection in an intensive care setting. Diagn Microbiol Infect Dis 76, 266–271 23711530 10.1016/j.diagmicrobio.2013.03.027

[B10] Dellinger R, Levy M, Rhodes A, et al. (2013). Surviving sepsis campaign: International guidelines for management of severe sepsis and septic shock. Crit Care Med 41, 580–637 23353941 10.1097/CCM.0b013e31827e83af

[B11] Gohil S, Cao C, Phelan M, et al. (2016). Impact of policies on the rise in sepsis incidence, 2000–2010. Clin Infect Dis 62, 695–703 26787173 10.1093/cid/civ1019 PMC4772840

[B12] Grapov D, Fahrmann J, Wanichthanarak K, and Khoomrung S (2018) Rise of deep learning for genomic, proteomic, and metabolomic data integration in precision medicine. OMICS 22, 630–636 30124358 10.1089/omi.2018.0097 PMC6207407

[B13] Harrison M, and Collins C (2015). Is procalcitonin-guided antimicrobial use cost-effective in adult patients with suspected bacterial infection and sepsis? Infect Control Hosp Epidemiol 36, 265–272 25695167 10.1017/ice.2014.60

[B14] Heyland D, Johnson A, Reynolds S, and Muscedere J (2011). Procalcitonin for reduced antibiotic exposure in the critical care setting: A systematic review and an economic evaluation. Crit Care Med 39, 1792–1799 21358400 10.1097/CCM.0b013e31821201a5

[B15] Kip M, Kusters R IJ zerman M, and Steuten L (2015). A PCT algorithm for discontinuation of antibiotic therapy is a cost-effective way to reduce antibiotic exposure in adult intensive care patients with sepsis. J Med Econ 18, 944–953 26105574 10.3111/13696998.2015.1064934

[B16] Linscheid P, Seboek D, Nylén E, et al. (2003). In vitro and in vivo calcitonin I gene expression in parenchymal cells: A novel product of human adipose tissue. Endocrinology 3, 144 10.1210/en.2003-0854 12960010

[B17] Mewes J, Pulia M, Mansour M, Broyles M, Nguyen H, and Steuten L (2019). The cost impact of PCT-guided antibiotic stewardship versus usual care for hospitalised patients with suspected sepsis or lower respiratory tract infections in the US: A health economic model analysis. PLoS One 14, e0214222 31013271 10.1371/journal.pone.0214222 PMC6478294

[B18] Müller B, White J, Nylén E, Snider R, Becker K, and Habener J (2001). Ubiquitous expression of the calcitonin-i gene in multiple tissues in response to sepsis. J Clin Endocrinol Metab 86, 396–404 11232031 10.1210/jcem.86.1.7089

[B19] Müller F, Christ-Crain M, Bregenzer T, et al. (2010). Procalcitonin levels predict bacteremia in patients with community-acquired pneumonia: A prospective cohort trial. Chest 138, 121–129 20299634 10.1378/chest.09-2920

[B20] Neumann P, Ganiats T, Russel L, Sanders G, Siegel J (Editors). (2016). Cost-Effectiveness in Health and Medicine. Oxford: Oxford University Press

[B21] Póvoa P, and Salluh J (2012). Biomarker-guided antibiotic therapy in adult critically ill patients: A critical review. Ann Intensive Care 2, 32 22824162 10.1186/2110-5820-2-32 PMC3475044

[B22] Schuetz P, Balk R, Briel M, et al. (2015). Economic evaluation of procalcitonin-guided antibiotic therapy in acute respiratory infections: A US health system perspective. Clin Chem Lab Med 53, 583–592 25581762 10.1515/cclm-2014-1015

[B23] Schuetz P, Christ-Crain M, Thomann R, et al. (2009). Effect of procalcitonin-based guidelines vs standard guidelines on antibiotic use in lower respiratory tract infections: The ProHOSP randomized controlled trial. JAMA 302, 1059–1066 19738090 10.1001/jama.2009.1297

[B24] Schuetz P, Chiappa V, Briel M, and Greenwald J (2011). Procalcitonin algorithms for antibiotic therapy decisions: A systematic review of randomized controlled trials and recommendations for clinical algorithms. Arch Intern Med 171, 1322–1331 21824946 10.1001/archinternmed.2011.318

[B25] Shapiro N, Wolfe R, Wright S, Moore R, and Bates D (2008). Who needs a blood culture? A prospectively derived and validated prediction rule. J Emerg Med 35, 255–264 18486413 10.1016/j.jemermed.2008.04.001

[B26] Singh N, Rogers P, Atwood C, Wagener M, and Yu V (2000). Short-course empiric antibiotic therapy for patients with pulmonary infiltrates in the intensive care unit. A proposed solution for indiscriminate anbitotic prescription. Am J Respir Crit Care Med 162, 505–511 10934078 10.1164/ajrccm.162.2.9909095

[B27] The Center for Disease Dynamics, Economics and Policy. (2017). ResistanceMap. Washington DC and New Delphi: CDDEPP

[B28] The Henry J. Kaiser Family Foundation. (2017). State Health Facts. Washington DC: KFF https://www.kff.org/statedata Accessed 926, 2017

[B29] van der Maas M, Kip M, Mantjes G, and Steuten L (2015). A procalcitonin algorithm used in adult ICU patients with sepsis saves costs by reducing antibiotic resistance and *C. difficile* infections. In: ISPOR 18th annual European Congress, 117–11, 2015, Milan, Italy

[B30] Wenzel R, and Edmond M (2000). Managing antibiotic resistance. N Engl J Med 343, 1961–1963 11136269 10.1056/NEJM200012283432610

